# A Study of the Key Factors on Production of Graphene Materials from Fe-Lignin Nanocomposites through a Molecular Cracking and Welding (MCW) Method

**DOI:** 10.3390/molecules27010154

**Published:** 2021-12-28

**Authors:** Qiangu Yan, Timothy Ketelboeter, Zhiyong Cai

**Affiliations:** 1 Ligwood LLC, Madison, WI 53705-2828, USA; yanqiangu@gmail.com; 2 Forest Products Lab, USDA Forest Service, Madison, WI 53726-2398, USA; timothy.ketelboeter@usda.gov

**Keywords:** graphene materials, Fe-lignin nanocomposite, catalytic graphitization, few-layer encapsulated metal nanoparticles, molecular cracking and welding (MCW) method, process variables, temperature, heating time, particle size

## Abstract

In this work, few-layer graphene materials were produced from Fe-lignin nanocomposites through a molecular cracking and welding (MCW) method. MCW process is a low-cost, scalable technique to fabricate few-layer graphene materials. It involves preparing metal (M)-lignin nanocomposites from kraft lignin and a transition metal catalyst, pretreating the M-lignin composites, and forming of the graphene-encapsulated metal structures by catalytic graphitization the M-lignin composites. Then, these graphene-encapsulated metal structures are opened by the molecule cracking reagents. The graphene shells are peeled off the metal core and simultaneously welded and reconstructed to graphene materials under a selected welding reagent. The critical parameters, including heating temperature, heating time, and particle sizes of the Fe-lignin composites, have been explored to understand the graphene formation mechanism and to obtain the optimized process parameters to improve the yield and selectivity of graphene materials.

## 1. Introduction

The challenges of sustainable development have driven people to find facile, environmentally friendly ways to produce carbon-based materials. Biomass presents an abundant and low-cost source of carbon. However, there have been limited studies on the use of wood or agricultural biomass as the carbon source to produce graphene-based materials. Lignin is the second major component of lignocellulosic biomass and the most abundant aromatic biopolymer resource on Earth [1]. More than 70 million metric tons of lignin are produced annually as a byproduct from wood delignification and pulping processes [2]. The kraft process is the most widely used pulping process and kraft lignin accounts for more than 90% of the world’s chemical pulp lignin production [3,4]. However, only ~1 to 2% of lignin is isolated from wood pulp for commercial applications, the majority is burned onsite for heat and pulping chemical recovery [5]. With the increasing awareness of environmental issues and the depletion of fossil fuels, there is a tremendous global research interest in utilizing biomass like lignin to produce sustainable/renewable fuels and chemicals [6] and carbon-based nanomaterials like active carbons [7,8], carbon fibers [9,10], templated carbon [11,12], graphene [13,14,15], and carbon foams [16] through thermal conversion technologies.

A batch type manufacturing process for converting kraft lignin to graphene materials has been developed by FPL and Domtar [17]. The method of synthesizing graphene-based materials involves preparing M-lignin composites from kraft lignin and a transition metal catalyst, pretreating the M-lignin composites, forming carbon-encapsulated metal structures from the M-lignin composites, forming of the graphene-encapsulated metal structures by thermally treating the M-lignin composites, and forming graphene materials through a molecular cracking and welding (MCW) method. MCW technique is a production process with two stages. In the first stage, biomass materials are catalytically graphitized to form graphene-encapsulated metal nanostructures (GEMNs). Then, in the second stage, these graphene-encapsulated metal structures are opened by the ‘molecule cracking reagents’ such as H_2_, H_2_O, CO_2_, and CH_4_. The graphene shells are peeled off the metal core and simultaneously welded and reconstructed to graphene materials under a selected welding reagent. The graphene shells will be joined through the unsaturated bonds of the carbon atoms on the edges, linked by carbon atoms from decomposed welding molecules, or agglomerate through van der Waals forces [15] under high temperature with selected welding reagent gases such as light hydrocarbons (methane, ethane, propane, natural gas, etc.) and hydrogen. Graphene materials, like graphene nanoplatelets, few-layer graphene sheets, and fluffy graphene can be produced through varying preparation conditions [15]. Applications of these graphene materials include advanced composites [18], sensors [19], electronics [20], fuel cells [21], solar cells [22], capacitors [23], batteries [24], thermal management applications [25], electronic display materials [26], inks and 3D-printer materials [27], barriers and films [28], etc.

It has been reported that the graphitization of solid carbon-based species is mainly affected by the heat treatment temperature [14], while other heat treatment variables like residence time and heating rate have a slight effect on the degree of graphitization of the products [14,15]. It was claimed that ambient gas phase has a relatively large effect on graphitization of solid carbon [14]. Different processing gases have been used to investigate the production of graphene materials from kraft lignin [14,15]. These gases serve as cracking/welding reagents and are selected from Ar, CH_4_, H_2_, CO_2_, and their mixtures to examine processing gas effects on graphene formation and product component distributions [14]. In addition to the cracking and welding gas composition, the conditions under which the graphene-encapsulated metal nanoparticles are exposed to the gas composition include the flow rate of the gas(es), the cracking/welding temperature, the heating rate to achieve the selected temperature, the reaction time, and the particle size of the GEMNs. These parameters may be selected to achieve a desired morphology and yield of the graphene material. This work focuses on studying how the process conditions including different heating temperatures, heating time, and GEIN grain particle size (i.e., the particle size of the carbon matrix embedded with GEINs) affect graphene material yield and structure. The optimized process conditions will help to design and operate the scale-up production process.

## 2. Experimental

### 2.1. Chemicals and Materials

Iron (III) nitrate nonahydrate (Fe(NO_3_)_3_·9H_2_O) and tetrahydrofuran were purchased from Sigma-Aldrich (St Louis, MO, USA). Kraft lignin (BioChoice) was supplied by Domtar Inc. (Plymouth, NC, USA).

### 2.2. Preparation of Iron (Fe)-Lignin Nanocomposites

The Fe-lignin nanocomposites with different transition metals were prepared by the coprecipitation method in a chemical fume hood as described previously [29]. To produce the iron-lignin composite, 82 g of iron (III) nitrate nonahydrate was dissolved in 50 mL DI water and stirred at 45 °C for 30 min. The iron nitrate solution was then added to kraft lignin solution (100 g kraft lignin in 150 mL tetrahydrofuran) and stirred energetically. The mixture was dried in the hood at room temperature for one week, and the sample was described as Fe-lignin.

### 2.3. Stabilization of Fe-Lignin Composites

It has been reported that Fe-lignin composites are thermally unstable, and an explosive thermal runaway may occur when heating the samples [30]. Therefore, the naturally dried Fe-lignin sample was first thermally decomposed in a muffle furnace before loading to a reactor for the catalytic graphitization process. Nitrogen gas was first introduced into the furnace at a flow rate of 100 mL/min for 30 min. The furnace temperature was increased to 300 °C at a rate of 2.5 °C/min and kept at 300 °C for 0.5 h. The furnace was cooled to ambient temperature naturally. Then, the decomposed sample was loaded into a ball mill machine and ground at 1000 rpm for 10 min.

### 2.4. Catalytic Graphitization of Fe-Lignin Composites to Graphene-Encapsulated Iron Nanoparticles (GEINs)

Fifty grams (50 g) of the decomposed Fe-lignin composites were each packed in the middle of a 2-inch OD ceramic tubular reactor. The carrier gas argon (99.99% purity) was first introduced into the reactor at a flow rate of 100 mL/min for 30 min. The reactor was heated at a temperature-programmed rate of 10 °C/min to a carbonization temperature (500, 600, 700, 800, 900, and 1000 °C) and kept at the carbonization temperature for 1 h. The furnace was cooled down by 10 °C/min to room temperature under an argon flow. An online Hiden QGA quantitative gas analysis system (Hiden Analytical, Livonia, MI, USA) was used to measure gaseous products during the graphitization process. The signals from the mass spectra of 2, 16, 28, and 44 (*m*/*z*) were identified as the main gaseous products H_2_, CH_4_, CO, and CO_2_, respectively.

### 2.5. Production of Graphene Nanomaterials through the Molecular Cracking and Welding (MCW) Process

Effect of reaction temperature: Fifty grams (50 g) of the GEIN sample were packed in the middle of a 2-inch OD ceramic tubular reactor. Argon (99.9% purity) was first introduced into the reactor at a flow rate of 50 mL/min for 30 min. Then, 80 mL/min methane was co-fed into the reactor and the reactor was heated to MCW temperature (700, 800, 900, and 1000 °C) with a heating rate of 10 °C/min and kept at the heating temperature for 1 h. The vent gas from the reactor was monitored using the online QGA system.

Effects of GEIN grain particle sizes: The GEIN grains were separated into different sizes: ≤50, 50–150, 150–250, and 500–700 μm. Fifty grams (50 g) of each sample were packed in the middle of a 2-inch OD ceramic tubular reactor in each run. The welding gas was introduced into the reactor. The reactor was temperature-programmed with a heating rate of 10 °C/min to 1000 °C and kept at 1000 °C for 1 h. The furnace was cooled down by 10 °C/min to room temperature.

Effects of reaction time: Fifty grams (50 g) of GEINs (150–250 μm) were packed in the middle of a 2-inch OD ceramic tubular reactor in each run. The welding gas was introduced into the reactor. The reactor was temperature-programmed with a heating rate of 10 °C/min to 1000 °C and kept at 1000 °C for 0.5, 1, 3, or 5 h. The furnace was cooled down by 10 °C/min to room temperature.

The yield of graphene product was calculated by

Y% = (M_1_/M_0_) × 100% (1)

where M_1_ was the carbon in the MCW sample, and M_0_ was the weight of GEIN sample before MCW. The solid carbon in the MCW sample was measured by TGA: Twenty milligrams of the MCW sample (20 mg) was put on a ceramic sample pan for TPO analysis in a Shimadzu TGA-50H instrument (Shimadzu TGA-50H, Columbia, MD, USA). The sample was heated in high flow of air (100 mL/min) from room temperature to 800 °C at a heating rate of 10 °C/min, and the weight change was recorded as related to carbon burning.

### 2.6. Characterization

The surface area of the thermally decomposed Fe-lignin composites was carried out using an automatic adsorption unit (Autosorb–1, Quantachrome, Boynton Beach, FL, USA). The samples were degassed at 300 °C for 5 h prior to analysis to remove any adsorbed moisture or other impurities bonded to the surface of the sample. X-ray powder diffraction (XRD) patterns of thermally treated iron-promoted lignin mixtures were obtained using a Rigaku Ultima III X-ray Diffraction System (Rigaku, The Woodlands, TX, USA) operated at 40 kV and 44 mA using Cu-Kα radiation with a wavelength of 1.5406 Å from 15° to 80° at a scan rate of 0.02 °s^−1^. The Jade powder diffraction analysis software from Materials Data, Inc. (Livermore, CA, USA) was used for both qualitative and quantitative analyses of polycrystalline powder materials. The mean particle size, L, was calculated for the most intense diffraction peaks of α-Fe, γ-Fe, and Fe_3_C using the Scherrer formula. The morphology of a thermally treated iron-promoted lignin mixture was investigated with a scanning electron microscope (SEM, Peabody, MA, USA). All SEM samples were precoated with 10-nm Pt before being introduced into the vacuum chamber. The system was operated with accelerating voltage of 10 kV. The particle sizes of thermally treated iron-promoted lignin mixtures were examined with a JEOL JEM-100CX II transmission electron microscope (TEM, Peabody, MA, USA) operated at accelerating voltage of 200 kV. All TEM samples were sonicated in ethanol solution for 1 min before being transferred to copper grids. Raman spectra of thermally treated iron-promoted lignin mixtures were obtained on a Jobin-Yvon microspectrometer (Edison, NJ, USA) equipped with an excitation laser source emitting at 514 nm and an incident power around 1 mW on a thin surface. Twenty spectra were collected for each sample. Deconvolution of the spectra was performed with the assumption of mixed Gaussian/Lorentzian peaks describing both the main D- and G-bands and the two minor ones. The D, G, and 2D peaks were fitted with Lorentz functions. The *A*_D_/*A*_G_ ratio was calculated using the integrated area of D to G peaks, which is related to the graphitic degree of graphene materials.

## 3. Results and Discussion

### 3.1. Formation of Graphene-Encapsulated Iron Nanoparticles (GEINs)

GEINs are the main product in the solid residues of catalytically graphitized biomass when iron is used as the transition metal [15]. Two possible mechanisms have been proposed to explain the formation of ordered carbon materials from solid carbon resources over transition metal catalysts: the dissolution–precipitation theory [31,32] and the metal carbide formation–decomposition process [33,34]. In the dissolution–precipitation theory, the carbon atoms in the disorder carbons diffuse and dissolve into the metal and/or metal carbide with elevated temperature. At a certain temperature, carbon atoms will be saturated in the selected metal, and as the temperature drops, carbon becomes supersaturated in the metal. The extra carbon atoms then precipitate from the metal and present as graphitic materials, since graphite is highly ordered carbon with the lowest Gibbs free energy [35,36]. In the metal carbide formation–decomposition process, amorphous carbon first reacts with metals and forms metal carbides under heating treatment. When the heating temperature is high enough, some carbide becomes unstable and decomposes to metal and graphite. Heating temperature is the most important factor in catalytic conversion of solid carbon to graphitic materials.

**Figure 1:**
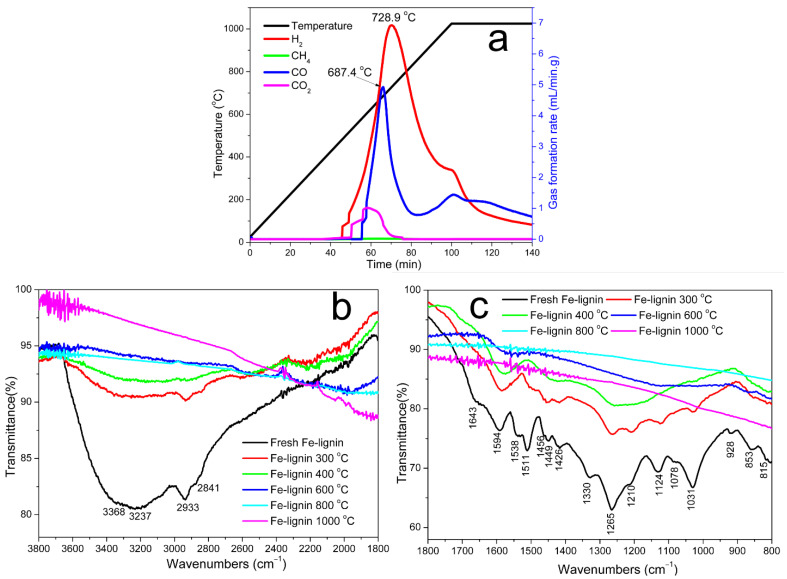
Gas evolution profiles (**a**) of temperature-programmed carbonization of the stabilized Fe-lignin composites under argon atmosphere and FTIR spectra (**b**,**c**) of the Fe-lignin composite thermally treated at different temperatures: 300 °C, 400 °C, 600 °C, 800 °C, and 1000 °C, respectively.

In the Fe-lignin composites, iron ions were uniformly distributed throughout lignin molecules by chelating to the oxygen-containing functional groups [37]. These Fe-lignin composites are thermally unstable and are decomposed to lignin char embedded with nano iron oxide particles when heated at 200–300 °C [37,38]. At elevated temperatures, the lignin char was catalytically carbonized, and alien elements, oxygen, and hydrogen in the lignin char were eliminated as gaseous water, carbon monoxide, carbon dioxide, and hydrogen (Figure 1a). The reducing gas reagents (hydrogen, CO, etc.) diffused into the carbonaceous matrix and reacted with the metal oxide (e.g., iron oxide) nanoparticles distributed within to form metallic catalyst particles during the catalytic graphitization process. Iron is known to be an excellent catalyst for cleaving carbon–carbon and carbon–hydrogen bonds [39]. Iron nanoparticles act as a catalyst to accelerate carbonization/graphitization of the char matrix, producing large amounts of gases and volatiles [13]. Meanwhile, under high heating temperatures, the disorder carbons around iron particles tend to diffuse and dissolve into the iron. Metallic iron is converted to different iron phases (α-Fe, γ-Fe, and Fe_3_C) with different carbon contents (Figure 1a). Iron nanoparticles reached a point of carbon saturation after a certain time period under the heating treatment temperature. When the temperature decreased, the iron particles saturated with the disordered carbon became supersaturated with carbon. Subsequently, carbon reprecipitates in the form of graphite crystals and the atoms deposit and rearrange on the surface of iron nanoparticles and form GEINs.

#### Effect of Temperature on Catalytic Graphitization Kraft Lignin to GEINs

Quantitative analysis of gaseous products from temperature-programmed catalytic graphitization (TPCG) of Fe-lignin composite was performed by an online RGA (Hiden QGA with a mass range of 300 AMU). Figure 1a shows the formation of the main gas species during the TPCG of the stabilized Fe-lignin composite. The trends of the main gaseous products were different from those of the raw lignin and the Fe-lignin that was not pre-decomposed [40]. It has been reported previously that functional groups like carboxyl and anhydride in the phenylpropane side chains are related to the formation of CO_2_ and CO at temperatures below 500 °C. A trace amount of CO_2_ and CO were released in the temperature zone of 300–524 °C (Figure 1a), implying that iron ions catalytically promote the decomposition of lignin since most of these functional groups are completely decomposed below 300 °C in the stabilization stage. No methane was detected during the catalytic graphitization process to the stabilized Fe-lignin composite. A small amount of CO_2_ was generated at the temperature between 523.6 and 783.8 °C, which is attributed to the cleavage of ether groups [5,13]. Significant amounts of CO are observed to appear beginning at 573.9 °C with peak formation at 687.4 °C mainly due to the cleavage of aromatic bonded oxygens (i.e., methoxy and phenolic groups) and the secondary cracking of oxygenate volatiles and tars [5,13]. Methane formation from decomposition of kraft lignin is usually contributed by methoxy groups (O-CH_3_) and side aliphatic chains [13], which are also completely decomposed during the stabilization process. Hydrogen evolution starts at 475 °C with a maximum formation peak at 723.5 °C; H_2_ formation is contributed by elimination of hydrogen from C−H bonds in aliphatic CH_x_ (x = 1–3) and aromatic rings via catalytic dissociation [13].

Figure 1b,c shows the FTIR spectra of the Fe-lignin composite heated at different temperatures (i.e., 300 °C, 400 °C, 600 °C, 800 C, and 1000 °C). For the fresh Fe-lignin composite, the peaks at 3368 and 3237 cm^−1^ are related to OH stretching vibrations of alcoholic and phenolic hydroxyl groups (involved in the hydrogen bonds) in lignin and hydroxyl groups in Fe(OH)_3_, respectively. The band intensity of the OH groups reduced significantly when the Fe-lignin composite was heated to 300 °C and 400 °C, indicating a decrease of the free hydroxyl groups on the kraft lignin and decomposition of Fe(OH)_3_ by dehydration reactions. With a further increase of the temperature to 600 °C and above, the hydroxyl groups are completely lost from the sample. The band at 2933 is assigned to aromatic methoxyl groups and methyl and methylene groups of the side chains; the band at 2841 cm^−1^ is assigned to symmetric C–H stretching in the -CH_2_- and tertiary C–H groups. The intensity of these two bands reduces drastically after heating the sample to 300 °C and 400 °C, likely due to the decomposition of the methyl and methylene groups in the side chains. The bands at 1710, 1665, 1456, 1362, 1210, and 1078 cm^−1^ are related to the C=O and C–O groups in lignin, and they disappear after the sample is heated at 300 °C and above. Bands of nitrate groups at 1535 and 1330 cm^−1^ are observed to disappear completely when the sample is heated at 300 °C and above. The bands at 1594, 1511, 1426, 1265, 1124, and 1031 cm^−1^ are assigned to vibrations of aromatic rings, and the intensities of these bands decrease with the increase of the heating temperature up to 600 °C and completely disappear at 800 °C and above. The FTIR results in Figure 1b,c are in good agreement with the profile of gaseous products in Figure 1a.

**Figure 2:**
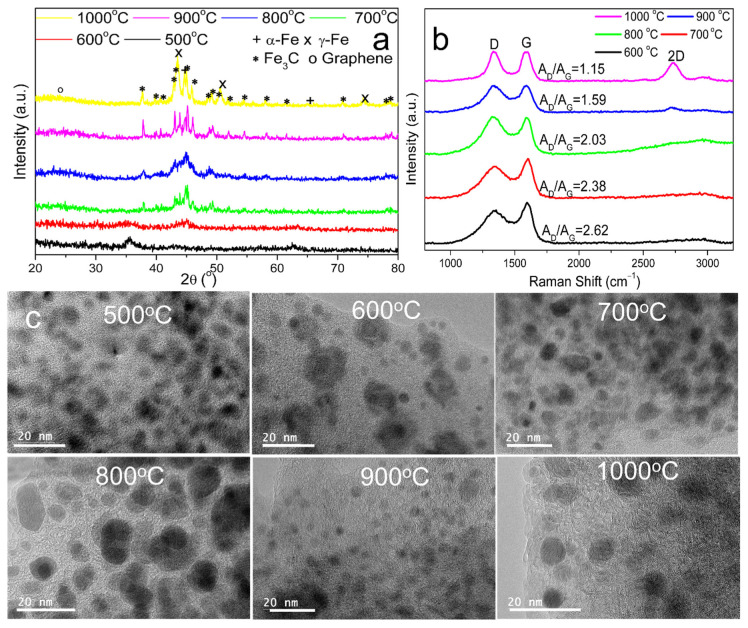
X-ray diffraction (XRD) patterns (**a**), Raman spectra (**b**), and HRTEM images (**c**) of Fe-lignin nanocomposites catalytically graphitized under argon atmosphere at different temperatures: 500 °C, 600 °C, 700 °C, 800 °C, 900 °C, and 1000 °C.

Figure 2a shows XRD patterns of the 10% Fe-lignin mixtures after thermal carbonization at different temperatures. Based on the XRD pattern, iron was reduced to FeO at 500 °C and was reduced to α-Fe in the lignin char matrix at 600 °C; these iron nanoparticles caught fire when the samples were exposed to air, since they were so active. When the temperature was increased to 700 °C, the intensity of the α-Fe diffraction peaks decreased while the γ-Fe increased, indicating α-Fe was partly transformed to γ-Fe. Cementite (Fe_3_C) phase formation was observed for the sample produced at 700 °C and above. Fe_3_C is related to carbon deposition from the carbonaceous gases [40] through the following reactions:

3Fe + CH_4_ → Fe_3_C + 2H_2_

3Fe + 2CO → Fe_3_C + CO_2_

The samples produced at 700 °C and above were stable in the air, possibly due to the iron particles being encapsulated in inert iron carbide and carbon shells. With further increase of the temperature to 800 °C, the Fe_3_C and γ-Fe diffraction peaks became more intense. As the temperature increased to 900 °C and 1000 °C, the material mainly showed the strong Fe_3_C diffraction peaks, which may be due to more carbon atoms diffused and dissolved into iron particles.

The crystallinity and graphitization degree can be further explained using *A*_D_/*A*_G_ values of Fe-lignin composites heated at various reaction temperatures. The calculated *A*_D_/*A*_G_ values were found to be 2.62, 2.38, 2.03, 1.59, and 1.15 at 600°C, 700 °C, 800 °C, 900 °C, and 1000 °C, respectively. Smaller *A*_D_/*A*_G_ values correlate with a higher graphitization degree. The decreased *A*_D_/*A*_G_ value with increasing reaction temperature indicates more of the well-ordered graphitic carbon is formed at a high temperature. Thus, in the present case, a higher graphitization degree was observed for the Fe-lignin composites carbonized at 1000 °C.

The microstructure and morphology of the Fe-lignin composites graphitized at temperatures between 500 and 1000 °C were investigated by HRTEM, and the images are illustrated in Figure 2c. Figure 2c shows the FeO nanoparticles (XRD results in Figure 2b) embedded in the amorphous lignin char matrix of the Fe-lignin-500 sample. The HRTEM image of the Fe-lignin-600 sample shows α-Fe nanoparticles (dark color) embedded in the amorphous carbon matrix (light color). The XRD results confirmed the formation of Fe_3_C and γ-Fe through the carbon atoms diffusion into α-Fe at 700 °C; these iron nanoparticles were encapsulated by carbon shells and embedded in the amorphous carbon matrix. No clear graphitic–carbon structure was observed in these carbon shells in samples heated up to 700 °C. One to two layers of graphitic carbon were observed to form around iron nanoparticles in the Fe-lignin composite carbonized at 800 °C, indicating the minimum temperature for the formation of GEINs was 800 °C; these GEINs were surrounded by amorphous carbon. The image of the Fe-lignin-900 sample showed similar GEIN structures formed with the Fe-lignin composite, where the graphitic carbon around iron nanoparticles increased to two to four layers, and the amorphous carbon was observed to convert to the turbostratic stacking structure [41]. When the carbonization temperature increased to 1000 °C, the graphitic shell increased to three to eight layers, and almost all the amorphous carbon was converted to the turbostratic stacking structure.

**Figure 3:**
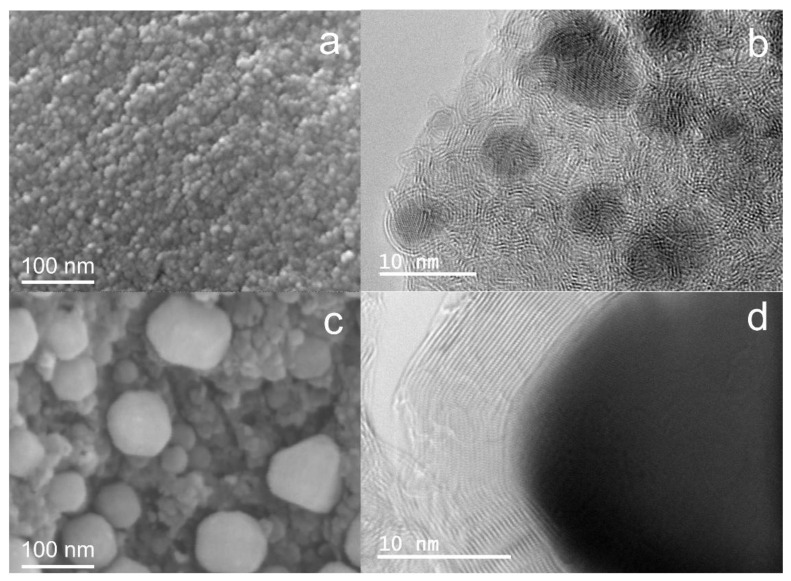
SEM and HRTEM images of GEINs embedded in the carbon matrix (**a**,**b**) and GEINs distributed on the outer surface of the carbon matrix (**c**,**d**).

Two forms of GEINs were observed in the graphitized Fe-lignin composites. The major GEINs (>90%) are nanoparticles with a uniform particle size of 3–10 nm (Figure 3a,b), have a 3–9-nm iron core with two to five layers of graphene shells (SEM and HRTEM results in Figure 3) surrounding it, and are embedded in turbostratic stacking graphitic structures. These GEINs are produced from the lignin char through the dissolution–precipitation process. Due to the carbon solubility in iron under the heating temperature, the graphene shells are limited to two to five layers.

In addition to the majority smaller size GEINs, larger nanoparticles distributed over the surface of the GEIN grains having diameters ranging from 10 to 100 nm (SEM and HRTEM results of Figure 3c,d) with the iron core encapsulated by 10–30 layers of concentric graphitic shells were also observed. These large-sized GEINs are formed from carbonaceous gases (CO and CH_4_) through a catalytic vapor decomposition (CVD) process [15,42]. During the graphitization process, lignin is first catalytically pyrolyzed to porous char structures [13,31]. Iron oxide nanoparticles on the surface of the pores in the lignin char are first reduced by reducing gaseous products. These surface iron particles have high mobility and will migrate on the lignin char surface and merge into larger size particles. Volatile carbonaceous gases (CO, light hydrocarbons, aromatics, and polycyclic aromatic hydrocarbons (PAHs)) generated from the decomposition of lignin [5] can adsorb, crack, and deposit through the CVD process to form graphitic materials over the surface of the iron particles:

CH_4_ → C_g_ + 2H_2_

2CO → C_g_ + CO_2_

**Scheme 1:**
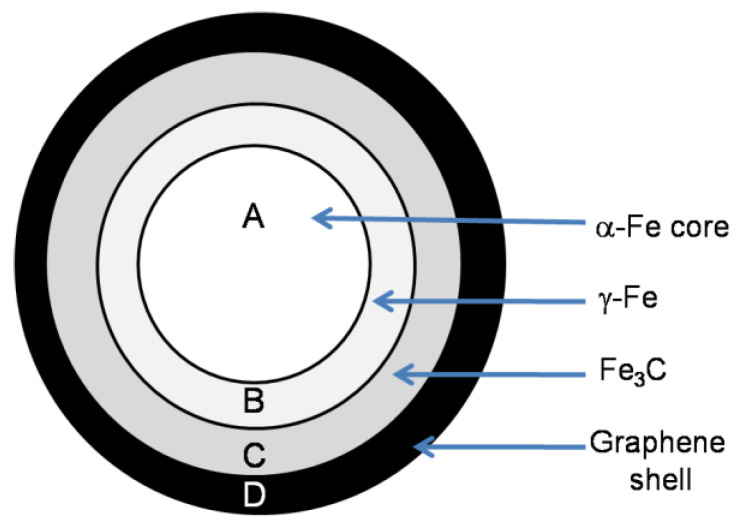
Illustration of the α-iron core/γ-iron/cementite (Fe_3_C)/few-layer graphene shell nanoparticle formed by catalytic graphitization of Fe-lignin nanocomposites.

Highly stable GEINs were produced by catalytic graphitization of Fe-lignin composites at high temperatures. The nanoparticle model was created based on the SEM, HRTEM, and XRD results. This model has an α-iron core, with a γ-iron phase layer, a carbide interface layer, and an outer graphene shell. The α-iron core/γ-iron/cementite/graphene shell nanocomposite is illustrated in Scheme 1.

### 3.2. Breaking of the GEINs by Cracking Reagents and Peeling the Graphene Shells off the Iron Cores

GEINs are the main product of catalytic graphitization of kraft lignin under inert atmospheres [14,15]. To produce graphene materials, GEINs are cracked by the selected cracking molecules at a high temperature [15]. The graphene shells on the outer layer of the GEINs are then peeled off the metal cores, and these cracked graphene shells serve as the building units to make different graphene-based materials.

**Scheme 2:**
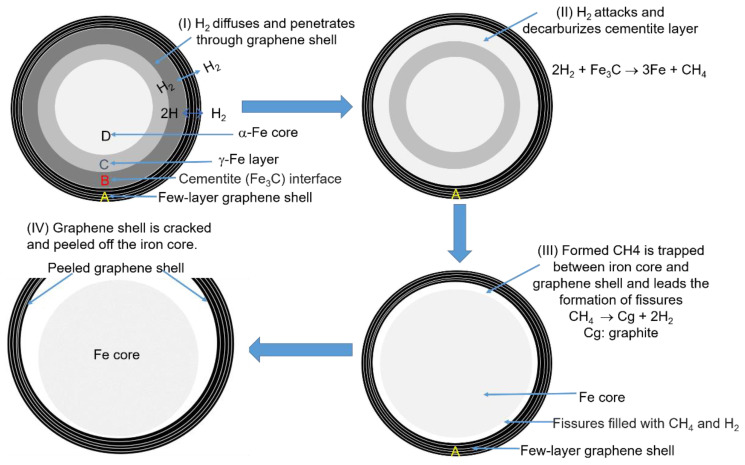
Scheme of possible cracking and peeling mechanism of GEINs by hydrogen through a heating process.

**Figure 4:**
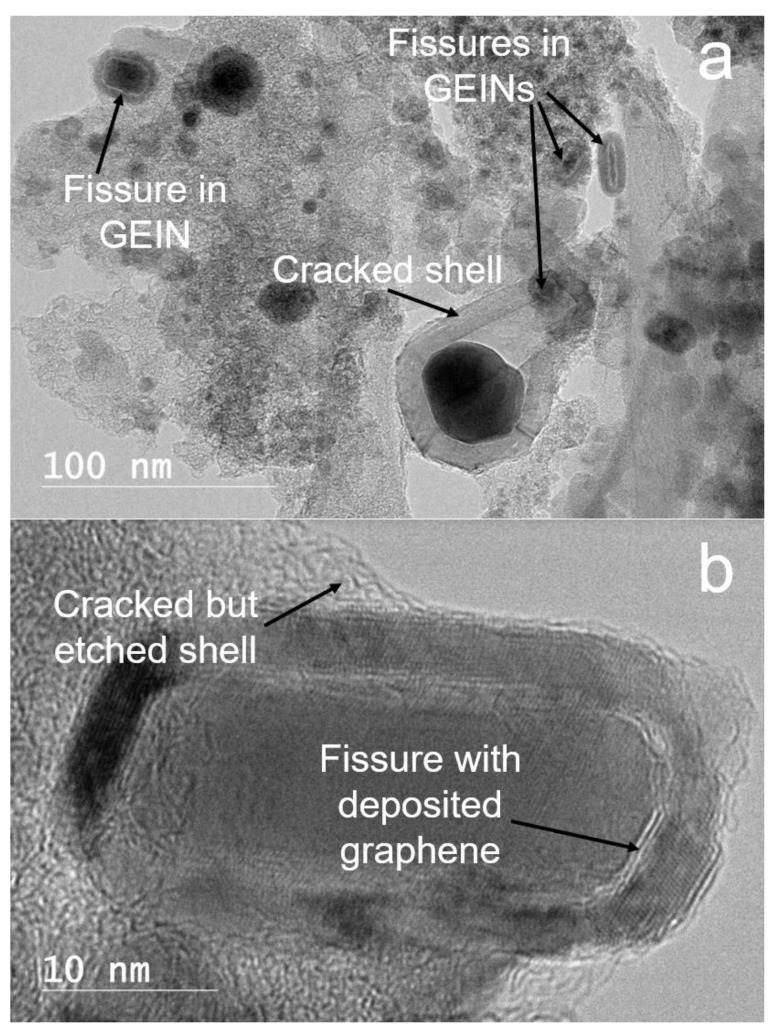
HRTEM images of GEIN sample (**a**) and a single GEIN (**b**) catalytically cracked by H_2_ at 900 °C.

Based on the structures of GEINs, it is reasonable to disintegrate the graphene shell and cementite (Fe_3_C) interface layer of GEINs using active reagents at a high temperature. Therefore, to break the GEINs, cracking molecules are used during the MCW process. Cracking molecules refer to reactive gaseous molecules that have the capability to react with the metal core (including iron carbide interface, γ-iron layer, and α-iron core for GEINs). H_2_O, CO_2_, O_2_, H_2_, and CH_4_ can react with the metal core [15,17]; however, most of the metal cores are encapsuled by 1–10 layers of graphene, and it is very difficult for H_2_O, CO_2_, O_2_, and CH_4_ to diffuse and penetrate through the few-layer graphene shell if there are no defective cracks. Fortunately, as the smallest molecule, H_2_ can easily diffuse and transport in solid materials like metals [43], alloys [44], and graphite [45]. Particularly, H_2_ is well-known to play the key role in the damage to steel and other metal alloys by the high temperature hydrogen attack (HTHA) [46]. To crack GEINs during the MCW process, hydrogen molecules diffuse and dissociate into the iron core and react with the dissolved carbon in the metal to form methane. Methane is a relatively large molecule, and it cannot diffuse out of the iron or graphite lattice structure. As a result, it will accumulate at the iron core (Fe)/graphene shell (C) boundaries to form fissures along the Fe-C interfaces and lead to crack formation. Figure 4 shows the HRTEM images of GEINs catalytically cracked by H_2_ at 900 °C; these images demonstrate the formation of fissures in the GEINs and the cracked GEINs. Compared to other active reagents, hydrogen is the best choice for the cracking molecule. Scheme 2 represents an illustration of the possible cracking mechanism of GEINs using hydrogen as the cracking reagent at an evolution temperature.

The activity of carbon in Fe_3_C was reported to be much higher than in the iron phases, since hydrogen reacts more rapidly with carbon in Fe_3_C than the carbon dissolved in γ-Fe [47]. Therefore, with the presence of hydrogen, the decarburization rate is greater in the case of cementite than that of α-Fe and γ-Fe. Carbon in alloys with iron is more stable with hydrogen than is cementite, which is an endothermic compound. Fe_3_C in the interface of the core–shell particles is first and quickly decarburized by hydrogen to iron phase and carbon is released as methane (CH_4_). While carbon in γ-iron is relatively stable compared to that of Fe_3_C, it is slowly decarburized to form methane. The pressure of the methane, which cannot diffuse from the iron layer formed from the decarburization of Fe_3_C, may exceed the cohesive strength of the iron core and cause interlayer fissuring between the original Fe_3_C and γ-iron layers. Within the fissures, part of the methane generated during decarburization is decomposed over surface iron atoms to graphene and hydrogen, explaining why there are two to three layers of graphene inside the iron cores:

CH_4_ (decarburized Fe_3_C and γ-iron) → C (graphene in fissuring) + 2H_2_

The cracking process of GEINs by hydrogen molecules may undergo the following steps: (I) hydrogen diffuses and penetrates through the graphene shell into the iron core and reacts with carbon inside Fe_3_C, γ-Fe and even α-Fe. (II) The diffused hydrogen molecule may dissociate into atomic hydrogen, then combine with local carbon atoms to form methane molecules. (III) Due to its larger size compared to atomic hydrogen, methane molecules from the decarburization process cannot diffuse through iron or graphitic structures and are trapped close to the reaction sites, leading to the build-up of methane pressure inside the GEIN structure. Increasing gas pressure inside solid particles promotes the extension of defects and appearance of fissures. Part of the methane generated during decarburization may be decomposed over surface iron atoms to form graphene and hydrogen. (IV) With time, methane pressure continues to increase at these fissures and causes cracks in the GEIN structure. The cracked graphene shell can then be peeled off the iron core.

Hydrogen also has strong etching effects on graphene structures by a gasification reaction at high temperatures. Methane is therefore selected as both the cracking and the welding reagent in this work, since methane can decompose to hydrogen gas and a carbon atom in the MCW process. H_2_ from methane decomposition will help to crack the GEINs and gasify the amorphous carbons in the grains of GEINs to form gaseous carbon-containing molecules, followed by re-deposition to form ordered graphene-based materials. The carbon atoms from methane decomposition will serve as an atomic glue to join the cracked graphene shells and can also deposit to vacancy or defect sites in the cracked graphene shells to improve the quality of the graphene product.

**Figure 5:**
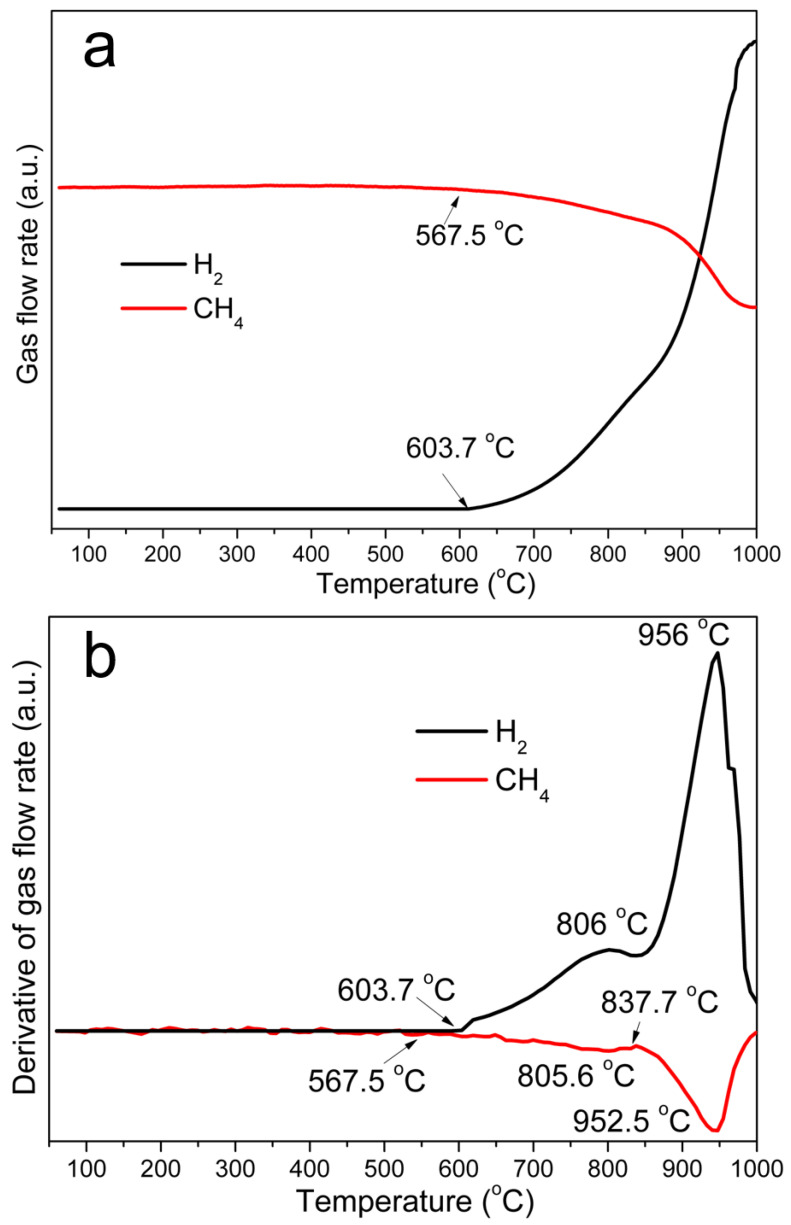
Flow rates (**a**) and their derivatives (**b**) of methane and hydrogen in the vent gas during the temperature-programmed MCW reaction. Feed gas: 50-mL/min Ar–80-mL/min CH_4_; heating rate, 10 °C/min; 50-g GEINs loaded to the reactor.

A temperature-programmed MCW reaction was performed by flowing an Ar-CH_4_ mixture (50-mL/min Ar80-mL/min CH_4_) through a fixed-bed reactor loaded with a 50-g GEIN sample. The reactor was heated to 1000 °C, with a heating ramp rate of 10 °C /min. The flow rates (Figure 5a) and their derivatives (Figure 5b) of methane and hydrogen in the vent gas are plotted in Figure 5. CH_4_ decomposition over GEINs was observed to initiate at 567.5 °C (Figure 5a), and only a small amount of methane was consumed at the heating temperature below 837.7 °C. Hydrogen production was initially detected at 603.7 °C, and a relatively low formation rate for H_2_ was obtained at temperatures under 837.7 °C. The methane consumption rate accelerated at 837.7 °C and reached the maximum at 1000 °C; the hydrogen formation rate also increased during this stage. Iron has been reported as an excellent metal component in catalysts for decomposition of methane; however, as shown in Figure 5, GEINs exhibited very poor activity in the decomposition of methane at low heating temperatures. This is because most of the iron particles are encapsulated in graphene shells, resulting in fewer available iron active sites for the adsorption and dissociation of methane molecules. Fortunately, there are some naked iron particles or GEINs with defects in their graphene shell. As the heating temperature increases to 567.5 °C, a small amount of methane will diffuse along the cracks of the GEIN graphene shell and adsorb onto the surface of the iron core, followed by the dissociation reaction of methane to hydrogen and carbon. The carbon atoms will deposit on the iron surface, while the hydrogen atoms may either combine and desorb as H_2_ to enter the gas phase or diffuse and dissolve into the iron core of the GEINs. As an excellent decarburization reagent, the dissolved hydrogen atoms then react with Fe_3_C and carbon in γ-Fe and α-Fe to form methane. With the increasing of the heating temperature, more methane decomposition reactions occur over GEINs, and consequently, more atomic hydrogen penetrates the core of the GEINs for the decarburization of Fe_3_C to methane. The methane accumulates at the interface between graphene shells and the iron core, causing the formation of fissures at the boundary; the trapped methane will erupt and break the outer graphene shell when its pressure is high enough. Conversely, the accumulated methane can re-dissociate to hydrogen and carbon, and the deposited carbon is assembled to the ordered graphite structure in the gaps. With more carbon deposited between the graphene shell and iron core, the iron core will expand in volume, facilitating the cracking of the graphene shell. As the graphene shell is cracked, the iron core will be exposed to the gaseous methane, and more iron active sites will be available for the decomposition of methane, which means the consumption rate of methane will be accelerated at this point. From the results in Figure 5, we can assume that most of the GEINs are cracked by the temperature of 837.7 °C, and the peeling process may be completed at around 950 °C, since both the changing rates of methane consumption and hydrogen formation (Figure 5b) reach their maximum at this point. The shell peeling and welding processes occur simultaneously with the promotion of methane decomposition as the heating temperature increases to 1000 °C.

### 3.3. Effect of Process Variables on Graphene Formation through MCW Process

The yield and the structures of the graphene products from GEINs in the MCW process are observed to depend on the process parameters (heating temperature, reaction atmosphere, and reaction time) and the particle size of the carbon matrix embedded with GEINs. The process conditions and the yields of the graphene materials are listed in Table 1.

#### 3.3.1. Effect of Heating Temperature on Formation of Graphene Materials through MCW Process

**Figure 6:**
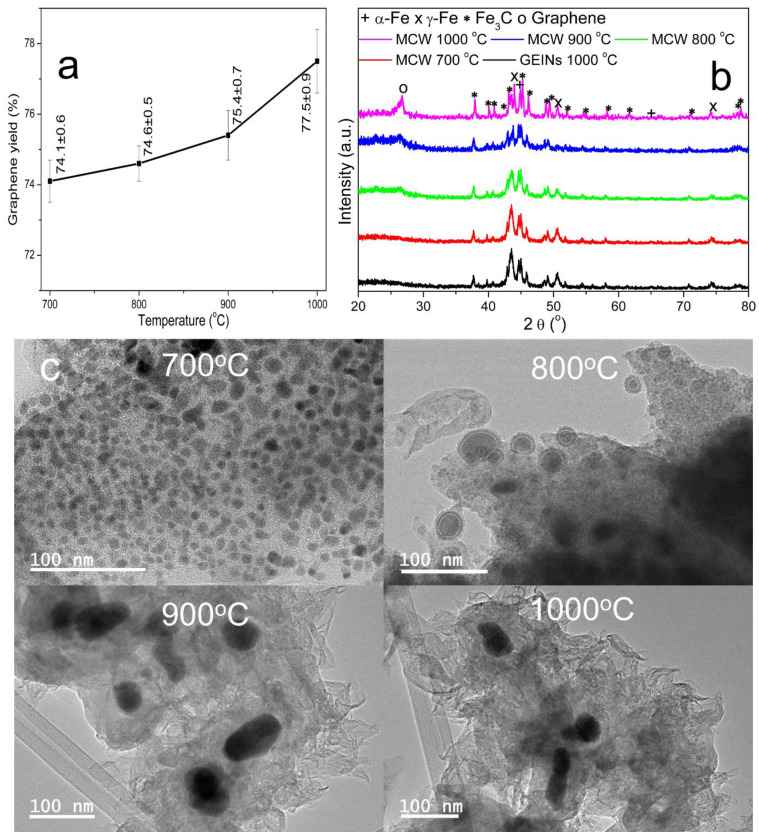
Yield of graphene materials (**a**). X-ray diffraction (XRD) patterns (**b**) and HRTEM images (**c**) of the cracked GEIN samples by methane molecules for 1 h at different reaction temperature of 700, 800, 900, and 1000 °C.

The effects of heating temperature on the yield of graphene-based materials from GEINs were evaluated in the range of 700–1000 °C and the results are plotted in Figure 6a. The yield of graphene materials increases with the increase of the MCW temperature in the temperature range tested, increasing from 74.1% at 700 °C to 77.5% at 1000 °C. The carbon content in the GEINs was measured as 73.5%; therefore, the increase of the graphene yield was contributed by the carbon atom from the decomposition of methane molecules.

XRD patterns of the graphene materials produced from GEINs under different heating temperatures are shown in Figure 6b. No noticeable changes in the XRD pattern are observed for the MCW sample at 700 °C compared to that of the original GEINs. When the temperature was increased to 800 °C, the intensities of both the α-Fe and γ-Fe diffraction peaks increased while the intensity of Fe_3_C peaks decreased, possibly due to cementite (Fe_3_C) in the outer layer of the iron core being decarburized and partially transformed to α-Fe and γ-Fe. Further increasing the temperature to 900 °C, α-Fe and γ-Fe peaks grow even sharper, while the Fe_3_C peaks continue to turn wider and weaker, indicating that the decarburization of Fe_3_C is ongoing in the metal cores of the GEINs. In addition to the α-Fe, γ-Fe, and Fe_3_C peaks, a diffraction peak at around 26.5° appears in the product materials, indicating the formation of graphene due to the MCW reaction process, where the graphene shells are cracked, peeled off from the iron cores, and welded into the graphene nanoplatelets. The stronger graphene diffraction peak when the temperature is increased to 1000 °C implies the graphene nanoplatelets are growing larger and thicker. The α-Fe, γ-Fe, and Fe_3_C peaks are also stronger and sharper for the 1000 °C sample, because after the GEINs are cracked and the graphene shells peeled off, the iron cores are now naked with high mobility and will migrate and sinter to large-sized iron particles. Methane can react with the naked iron particles to form iron carbide.

HRTEM images of the GEIN samples cracked by methane molecules for 1 h at different reaction temperatures (700, 800, 900, and 1000 °C) are shown in Figure 6c. There is no obvious change for the GEIN sample cracked at 700 °C, because the GEIN particles keep their original structures, and the graphene shells are not yet cracked at this point, i.e., the iron cores are still encapsulated in 1–10 layers of graphene shells (Figure 6c). At 800 °C, the HRTEM image shows that fissures are formed between the graphene shells and the iron cores of the GEINs due to the decarburization of Fe_3_C by hydrogen from the decomposed methane. When the temperature increases to 900 °C, the core–shell structures of the GEINs are cracked, and the graphene shells are skinned off the iron cores to form graphene nanoplatelets. The sizes of the iron particles significantly increased to 30–100 nm due to coalescence of the naked iron cores. HRTEM image of the cracked GEIN sample at 1000 °C demonstrates the continuation of the MCW process; more graphene materials are formed, and the iron particles are separated from the graphene structures.

#### 3.3.2. Effects of GEIN Grain Particle Sizes

**Figure 7:**
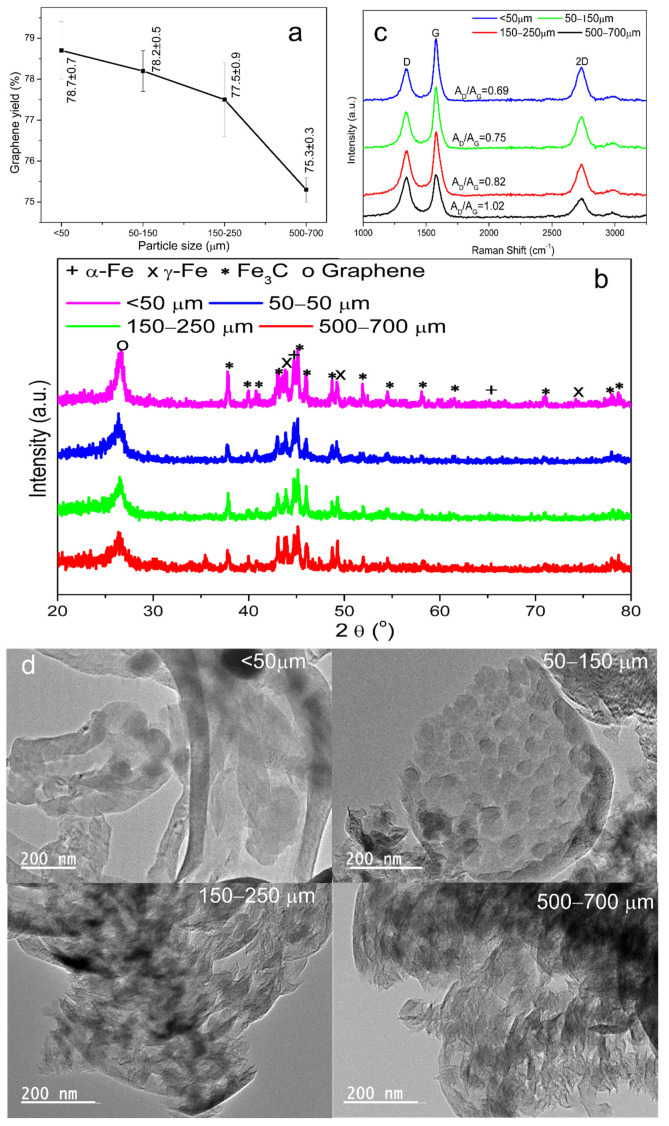
Yield of graphene materials (**a**), XRD patterns (**b**), Raman spectra (**c**), and HRTEM (**d**) images of the cracked GEIN samples from different GEIN grain particle sizes through MCW at 1000 °C.

The impact of the GEIN grain particle size on the MCW process was studied in the current work. Four initial GEIN grain particle size fractions: <50, 50−150, 150−250, and 500−700 µm were screened for studying the effect of particle size on the yield of graphene materials, which is illustrated in Figure 7a. It was shown that the GEIN grain particle size has a significant influence on the graphene product yield. The yield of graphene materials decreased from 78.7% to 76.1% as the particle size increased from fine powder (<50 µm) to large-sized grains (500−700 µm) (Figure 7a). The yields decrease with the increasing GEIN particle size because with a smaller particle size, a relatively larger specific surface area contacts the gas phase, the mass transfer resistance is negligible, and the MCW process may be under kinetic control; therefore, under the same reaction conditions, higher reaction rates and higher graphene yield are obtained over smaller particles. The MCW process mainly occurs in the surface of the larger particle grains and is affected by the mass and heat transfer. In larger particles, the mass and heat transfer resistance are greater, resulting in lower reaction rates inside the particle in which the MCW process takes place. This causes a slow reaction progress during cracking and welding, resulting in a low graphene yield and smaller pieces of graphene with fewer layers and more defects.

The XRD patterns of graphene materials prepared from GEIN precursors with different grain sizes are shown in Figure 7b. The peak at 2θ = 26.5° becomes sharp and tight when the GEIN grain size decreases from 500−700 µm to less than 50 µm, indicating the graphene nanoplatelets grow larger and thicker with a decrease in GEIN grain size. The diffraction peaks of α-Fe, γ-Fe, and Fe_3_C peaks also grow strong and narrow for smaller GEIN grains, possibly due to high mobility of the cracked graphene shells and the iron cores from the smaller particles. The peeled graphene shells and the naked iron particles from the smaller GEIN grains are the most mobile during the MCW process. Welding of the graphene nanoplatelets and sintering of the iron cores will be accelerated by the free movement. The graphene nanoplatelets can be jointed into larger and thicker pieces through all possible directions, while the iron cores will migrate and coalesce to large-sized iron particles. These large iron particles can react with methane to form iron carbide. Due to particle agglomeration in the large GEIN grains, the cracked graphene shells and the iron cores have relatively low mobility; therefore, the welding reactions of graphene shells and sintering of iron particles are limited during the MCW process and result in smaller and fewer layers of graphene materials.

The quality of the graphene materials was investigated using Raman spectroscopic measurements. As presented in Figure 7c, two distinct bands are observed. The D-band at 1345 cm^−1^ represents either defect or amorphous carbon, whereas the G-band at 1573 cm^−1^ represents the ordered graphene structures. The graphitization degree of the graphene materials is measured through the intensity ratio of *A*_D_/*A*_G_. *A*_D_/*A*_G_ had an increasing trend as the GEIN grain size increased from below 50 µm to 500−700 µm, confirming the lower graphitization degree of the larger GEIN particle samples.

Figure 7d displays HRTEM images of the cracked GEIN samples with different grain sizes by the MCW process. The images show that three-dimensional graphitic structures are the main products from the GEIN particles below 50 µm, and these graphitic structures are made up of few-layer graphene shells made from a welding process. The average thickness of the graphitic structures is around 5 nm. Dimensions in-plane of the graphene materials varies from hundreds of nanometers to a few microns. HRTEM images reveal graphene nanoplates are the main products of the GEINs with particle sizes of 50–150 and 150–250 µm. Graphene nanoplates are composed of several sheets of cracked graphene shells with an overall thickness of 1–3 nm and in-plane sizes of 200–500 nm. Graphene nanoplates from 50−150-µm particles are slightly larger and thicker than those from 150–250-µm particles. When GEIN grain sizes increases to 500–700 µm, fluffy graphene becomes the main structure in the MCW product. Fluffy graphene is a two-dimensional graphitic sheet. It is formed from the cracked graphene shells with fewer further welding reactions and is usually made up of one to five layers of graphene, has an average thickness of one nanometer or less, and has an in-plane size between 30 and 100 nm.

Overall, the experimental results in Figure 7 demonstrate that the yield, geometry, size, and thickness of the graphene materials decreased with an increase of the GEIN particle size.

#### 3.3.3. Effects of MCW Reaction Time

The reaction time is also an important parameter for the production of graphene from GEINs through the MCW process. To study the effect of the reaction time, it was varied 0.5, 1, 2, and 3 h while keeping the reaction temperature constant at 1000 °C; the reactant gas was 50-mL/min argon and 80-mL/min CH_4_.

**Figure 8:**
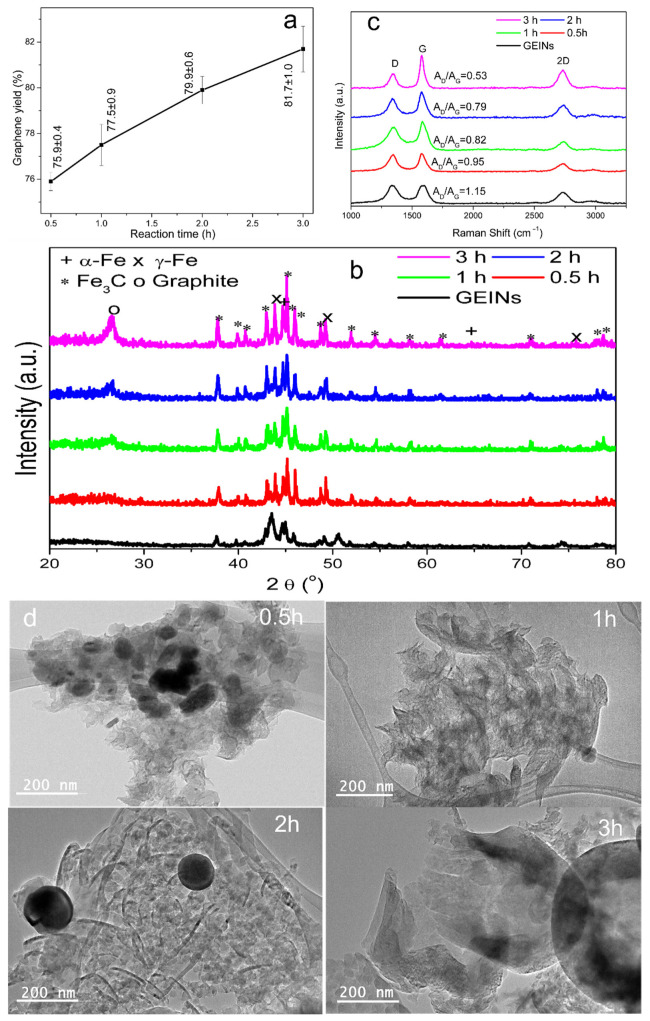
Yield of graphene materials (**a**), XRD patterns (**b**), Raman spectra (**c**), and HRTEM (**d**) images of the cracked GEIN samples using methane at 1000 °C with different reaction times.

The effects of reaction time on the yield of graphene materials are plotted in Figure 8a. The yield of graphene materials was 75.9% at the reaction time of 30 min. As the reaction time extended to 1 h, the yield increased to 77.5%. The yield further increased to 79.9% and 81.7% for the reaction times of 2 h and 3 h, respectively. Graphene yield increased with the increased reaction time, because more carbon atoms from methane can participate in the MCW process. As the molecular welding reagent in this work, methane molecules will decompose and provide atomic carbon to join the cracked graphene shells. Concurrently, H_2_ from methane decomposition can react with amorphous carbon in the GEIN grains to form gaseous carbon-containing molecules (e.g., CH_4_), followed by re-deposition to form graphene materials. Atomic carbon from methane can deposit in the voids or defects on the graphene materials to improve their quality. The extra methane in the flow can serve directly as a reactant to form graphene over the surface of the metal particles.

Figure 8b shows the XRD patterns of the products obtained at different reaction times (0.5, 1, 2, and 3 h). The relative intensity of the graphene diffraction peak (2θ = 26.5°) increases with the increased reaction time. A wide and flat diffraction peak at around 26.5° is observed for the graphene sample of 0.5 h. This peak becomes strong and sharp with the increased reaction time, indicating that graphene nanoplatelets grow with improved quality over a prolonged reaction time. The diffraction peaks of α-Fe, γ-Fe, and Fe_3_C peaks also grow sharp and tight with the longer reaction time, possibly due to sintering of the iron cores with the extended reaction.

The quality of graphene produced from GEINs at different reaction times was determined using the Raman spectroscopy analysis to assess the area ratio between bands D and G (*A*_D_/*A*_G_) (Figure 8c). The *A*_D_/*A*_G_ ratio decreased with the increasing reaction time, indicating a decrease in defects present in the graphene materials. The I_2D_/I_G_ ratio also decreases with the increasing reaction time, indicating more graphene layers are present in graphene samples produced with longer reaction times.

Figure 8d illustrates the HRTEM images of the graphene materials prepared for 0.5, 1, 2 and 3 h. After 0.5 h of the MCW process, fluffy graphene is produced from the cracked graphene shells, since fewer welding reactions occur between the cracked shells due to the short time process. The formation of fluffy graphene generally involves random distribution along various directions but fewer or no jointing occurs, and it is usually made up of one to five layers of graphene with an in-plane size of 30–80 nm. In the HRTEM image of the sample after 1 h of MCW reaction, large pieces of flake-like graphene with sizes of around 200–300 nm can be seen, suggesting that welding or jointing reactions happen between the cracked graphene shells. The welding action mainly involves bonding along the horizontal direction of the cracked graphene shell units. The HRTEM image reveals that graphene nanoplates are the main products of the GEINs after 2 h of reaction. These graphene nanoplates are jointed along the horizontal and the perpendicular directions of the cracked graphene shell units and demonstrate an overall thickness of 1–3 nm and in-plane size of 200–500 nm. When the MCW process runs for 3 h, the graphene materials are mainly composed of three-dimensional graphitic nanochips. These graphitic structures are produced by welding more cracked graphene shells along the horizontal and the perpendicular directions due to the prolonged reaction time. The graphitic nanochips show an in-plane size of 0.5–2 microns with average thickness of 5 nm.

## 4. Conclusions

Few-layer graphene materials were synthesized from lignin resources using the MCW method over a fixed-bed reaction system. This is a single-step process with two stages. In the first stage, few-layer graphene-encapsulated iron nanoparticles (GEINs) were formed through the catalytic graphitization of Fe-lignin nanocomposites. Then, in the second stage, these GEINs were cracked and opened into few-layer graphene shell-like structures, which were welded and reconstructed under high temperatures with selected welding reagent gases to form few-layer graphene materials (fluffy, chain, few-layer graphene nanoplatelets, flattened or curved sheet-like). The influences of heating temperature, heating time, and Fe-lignin composite particle size on the yield of few-layer nano-shell graphene materials produced from lignin resources were investigated. The ideal welding temperatures were found to be at least 1000 °C, with the heating time ranging from 0.5 to 1 h. The optimized Fe-lignin composite particle size was found to be between 150 and 250 μm.

## Figures and Tables

**Table 1:** Graphene-based materials from graphene-encapsulated iron nanoparticles (GEINs). The “@C” indicates the carbon (graphene) encapsulation.

GEMNs	GEMN Agglomerate Particle Size (µm)	Heating Temperature (°C)	Cracking-Welding Gas (mL/min)		Reaction Time (h)	Graphene Yield (%)
			Ar	CH_4_		
Fe@C	150–250	700	50	80	1	74.1 ± 0.6
Fe@C	150–250	800	50	80	1	74.6 ± 0.5
Fe@C	150–250	900	50	80	1	75.4 ± 0.7
Fe@C	150–250	1000	50	80	1	77.5 ± 0.9
Fe@C	150–250	1000	50	80	0.5	75.9 ± 0.4
Fe@C	150–250	1000	50	80	2	79.9 ± 0.6
Fe@C	150–250	1000	50	80	3	81.7 ± 1.0
Fe@C	<50	1000	50	80	1	78.7 ± 0.7
Fe@C	50–150	1000	50	80	1	78.2 ± 0.5
Fe@C	500–700	1000	50	80	1	75.3 ± 0.3

## Data Availability

Not applicable.

## References

[1] Zoghlami A., Paës G. (2019). Lignocellulosic Biomass: Understanding Recalcitrance and Predicting Hydrolysis. Front. Chem..

[2] Li T., Takkellapati S. (2018). The current and emerging sources of technical lignins and their applications. Biofuels Bioprod. Biorefin..

[3] Pérez A.D., Fiskari J., Schuur B. (2021). Delignification of Low-Energy Mechanical Pulp (Asplund Fibers) in a Deep Eutectic Solvent System of Choline Chloride and Lactic Acid. Front. Chem..

[4] Vásquez-Garay F., Carrillo-Varela I., Vidal C., Reyes-Contreras P., Faccini M., Mendonça R.T. (2021). A Review on the Lignin Biopolymer and Its Integration in the Elaboration of Sustainable Materials. Sustainability.

[5] Yan Q., Li J., Zhang J., Cai Z. (2018). Thermal Decomposition of Kraft Lignin under Gas Atmospheres of Argon, Hydrogen, and Carbon Dioxide. Polymers.

[6] Kucharska K., Rybarczyk P., Hołowacz I., Łukajtis R., Glinka M., Kamiński M. (2018). Pretreatment of Lignocellulosic Materials as Substrates for Fermentation Processes. Molecules.

[7] Hayashi J., Kazehaya A., Muroyama K., Watkinson A.P. (2000). Preparation of activated carbon from lignin by chemical activation. Carbon.

[8] Suhas P.J.M., Carrott M.M.L.R. (2007). Lignin—From natural adsorbent to activated carbon: A review. Bioresour. Technol..

[9] Poursorkhabi V., Abdelwahab M.A., Misra M., Khalil H., Gharabaghi B., Mohanty A.K. (2020). Processing, Carbonization, and Characterization of Lignin Based Electrospun Carbon Fibers: A Review. Front. Energy Res..

[10] Wang S., Bai J., Innocent M.T., Wang Q., Xiang H., Tang J., Zhu M. (2021). Lignin-based carbon fibers: Formation, modification and potential applications. Green Energy Environ..

[11] Ponomarev N., Sillanpaa M. (2019). Combined chemical-templated activation of hydrolytic lignin for producing porous carbon. Ind. Crop. Prod..

[12] Rosas J.M., Berenguer R., Valero-Romero M.J., Rodríguez-Mirasol J., Cordero T. (2014). Preparation of different carbon materials by thermochemical conversion of lignin. Front. Mater..

[13] Yan Q., Li J., Zhang X., Hassan E.B., Wang C., Zhang J., Cai Z. (2018). Catalytic graphitization of kraft lignin to graphene-based structures with four different transitional metals. J. Nanopart. Res..

[14] Yan Q., Zhang X., Li J., Hassan E.B., Wang C., Zhang J., Cai Z. (2018). Catalytic conversion of Kraft lignin to bio-multilayer graphene materials under different atmospheres. J. Mater. Sci..

[15] Yan Q., Li J., Zhang X., Zhang J., Cai Z. (2019). Mass production of graphene materials from solid carbon sources using a molecular cracking and welding method. J. Mater. Chem. A.

[16] Yan Q., Arango R., Li J., Cai Z. (2021). Fabrication and characterization of carbon foams using 100% Kraft lignin. Mater. Des..

[17] Cai Z., Yan Q., Zhang J., Li J., Marcoccia B.S., Freiberg J.D. (2020). Method for Synthesizing Graphene from Encapsulated Particles. U.S. Patent.

[18] Lalwani G., Henslee A.M., Farshid B., Lin L., Kasper F.K., Qin Y.-X., Mikos A.G., Sitharaman B. (2013). Two-Dimensional Nanostructure-Reinforced Biodegradable Polymeric Nanocomposites for Bone Tissue Engineering. Biomacromolecules.

[19] Schedin F., Geim A.K., Morozov S.V., Hill E.W., Blake P., Katsnelson M.I., Novoselov K.S. (2007). Detection of individual gas molecules adsorbed on graphene. Nat. Mater..

[20] Akinwande D., Tao L., Yu Q., Lou X., Peng P., Kuzum D. (2015). Large-Area Graphene Electrodes: Using CVD to facilitate applications in commercial touchscreens, flexible nanoelectronics, and neural interfaces. IEEE Nanotechnol. Mag..

[21] Fei H., Ye R., Ye G., Gong Y., Peng Z., Fan X., Samuel E.L.G., Ajayan P.M., Tour J.M. (2014). Boron- and Nitrogen-Doped Graphene Quantum Dots/Graphene Hybrid Nanoplatelets as Efficient Electrocatalysts for Oxygen Reduction. ACS Nano.

[22] Zhong M., Xu D., Yu X., Huang K., Liu X., Qu Y., Xu Y., Yang D. (2016). Interface coupling in graphene/fluorographene heterostructure for high-performance graphene/silicon solar cells. Nano Energy.

[23] Stoller M.D., Park S., Zhu Y., An J., Ruoff R.S. (2008). Graphene-Based Ultracapacitors. Nano Lett..

[24] Yao F., Güneş F., Ta H.Q., Lee S.M., Chae S.J., Sheem K.Y., Cojocaru C.S., Xie S.S., Lee Y.H. (2012). Diffusion Mechanism of Lithium Ion through Basal Plane of Layered Graphene. J. Am. Chem. Soc..

[25] Amini S., Garay J., Liu G., Balandin A.A., Abbaschian R. (2010). Growth of large-area graphene films from metal-carbon melts. J. Appl. Phys..

[26] Eda G., Fanchini G., Chhowalla M. (2008). Large-area ultrathin films of reduced graphene oxide as a transparent and flexible electronic material. Nat. Nanotechnol..

[27] Zhu C., Han T.Y.-J., Duoss E.B., Golobic A.M., Kuntz J.D., Spadaccini C.M., Worsley M.A. (2015). Highly compressible 3D periodic graphene aerogel microlattices. Nat. Commun..

[28] Gao W., Majumder M., Alemany L.B., Narayanan T.N., Ibarra M.A., Pradhan B.K., Ajayan P.M. (2011). Engineered Graphite Oxide Materials for Application in Water Purification. ACS Appl. Mater. Interfaces.

[29] Yan Q., Cai Z. (2020). Issues in Preparation of Metal-Lignin Nanocomposites by Coprecipitation Method. J. Inorg. Organomet. Polym. Mater..

[30] Yan Q., Boardman C.R., Cai Z. (2020). Thermal stability of metal-lignin composites prepared by coprecipitation method. Thermochim. Acta.

[31] Kicinski W., Dyjak S. (2020). Transition metal impurities in carbon-based materials: Pitfalls, artifacts and deleterious effects. Carbon.

[32] Bleu Y., Bourquard F., Tite T., Loir A.-S., Maddi C., Donnet C., Garrelie F. (2018). Review of Graphene Growth from a Solid Carbon Source by Pulsed Laser Deposition (PLD). Front. Chem..

[33] Oya A., Otani S. (1979). Catalytic graphitization of carbons by various metals. Carbon.

[34] Lin Q., Feng Z., Liu Z., Guo Q., Hu Z., He L., Ye H. (2015). Atomic scale investigations of catalyst and catalytic graphitization in a silicon and titanium doped graphite. Carbon.

[35] Amato G. (2018). High Temperature Growth of Graphene from Cobalt Volume: Effect on Structural Properties. Materials.

[36] Zhang H., Zhang X., Sun X., Ma Y. (2013). Shape-controlled synthesis of nanocarbons through direct conversion of carbon dioxide. Sci. Rep..

[37] Yan Q., Cai Z. (2020). Effect of Solvents on Fe-Lignin Precursors for Production Graphene-Based Nanostructures. Molecules.

[38] Yu H., Yang J., Shi P., Li M., Bian J. (2021). Synthesis of a Lignin-Fe/Mn Binary Oxide Blend Nanocomposite and Its Adsorption Capacity for Methylene Blue. ACS Omega.

[39] Bakas N.J., Neidig M.L. (2021). Additive and Counterion Effects in Iron-Catalyzed Reactions Relevant to C–C Bond Formation. ACS Catal..

[40] Kim D.-Y., Heo Y.-U., Sasaki Y. (2013). Cementite Formation from Magnetite under High Pressure Conditions. ISIJ Int..

[41] Zhang X., Yan Q., Hassan E.B., Li J., Cai Z., Zhang J. (2017). Temperature effects on formation of carbon-based nanomaterials from kraft lignin. Mater. Lett..

[42] Yan Q., Li J., Zhang X., Zhang J., Cai Z. (2018). In situ formation of graphene-encapsulated iron nanoparticles in carbon frames through catalytic graphitization of kraft lignin. Nanomater. Nanotechnol..

[43] Hempelmann R. (1984). Diffusion of hydrogen in metals. J. Less-Common Met..

[44] Fort D., Harris I.R. (1975). The physical properties of some palladium alloy hydrogen diffusion membrane materials. J. Less-Common Met..

[45] Atsumi H. (2002). Hydrogen bulk retention in graphite and kinetics of diffusion. J. Nucl. Mater..

[46] Benac D.J., McAndrew P. (2012). Reducing the Risk of High Temperature Hydrogen Attack (HTHA) Failures. J. Fail. Anal. Prev..

[47] Cheng G., Calizo I., Hacker C.A., Richter C.A., Hight Walker A.R. (2016). Fe-catalyzed etching of exfoliated graphite through carbon hydrogenation. Carbon.

